# Anti-GQ1b-Positive Miller Fisher Syndrome Following Pfizer Bivalent COVID-19 Vaccination

**DOI:** 10.7759/cureus.105119

**Published:** 2026-03-12

**Authors:** Abdul K Naasan, Hirah Sheikh, Tanya D Page

**Affiliations:** 1 Department of Research, Rocky Vista University College of Osteopathic Medicine, Parker, USA; 2 Chief of Physical Medicine and Rehabilitation, The Brooklyn Hospital Center, Brooklyn, USA

**Keywords:** anti-gq1b antibody, covid-19 vaccine, guillain-barré syndrome, intravenous immunoglobulin (ivig), miller fisher syndrome

## Abstract

Miller Fisher syndrome (MFS) is a rare, immune-mediated variant of Guillain-Barré syndrome characterized by acute onset of ataxia, areflexia, and ophthalmoplegia. It typically develops following an antecedent event, such as an infection or, in rare instances, a vaccination, which triggers the production of anti-ganglioside antibodies. While cases have been anecdotally linked to COVID-19 vaccination, serologically confirmed reports remain uncommon. We report a case of a 51-year-old, previously independent male who presented with acute neurological deterioration, including ascending numbness, severe ataxia, and diplopia, 10 days after receiving a Pfizer SARS-CoV-2 bivalent vaccine. Diagnostic workup confirmed the clinical diagnosis, with cerebrospinal fluid showing cytoalbuminologic dissociation and a positive serum anti-GQ1b antibody test. The patient was treated with a five-day course of intravenous immunoglobulin and subsequently underwent intensive inpatient rehabilitation, leading to a significant functional recovery. This case provides robust, serologically confirmed evidence of MFS following COVID-19 vaccination, highlighting a rare but treatable neurological complication. Clinicians should maintain vigilance for this syndrome in patients presenting with acute cranial nerve and cerebellar deficits after a recent immunological stimulus.

## Introduction

Miller Fisher syndrome (MFS) is a rare, immune-mediated polyneuropathy recognized as a localized variant of Guillain-Barré syndrome (GBS). It is classically defined by the clinical triad of acute-onset ophthalmoplegia (paralysis of eye muscles), ataxia (loss of voluntary muscle coordination), and areflexia (absence of deep tendon reflexes) [1]. The underlying pathophysiology is attributed to an autoimmune response following an antecedent event, such as an infection or, rarely, a vaccination. This process leads to the generation of specific autoantibodies, most notably against the ganglioside GQ1b, which is highly concentrated in the oculomotor, trochlear, and abducens nerves, as well as in the dorsal root ganglia [2]. This targeted autoimmune attack directly correlates with the syndrome's distinct clinical features.

While the most common trigger for MFS is infection, other immunological stimuli have been implicated. The global COVID-19 pandemic and subsequent mass vaccination campaigns have brought new attention to rare neuro-immunological phenomena. Cases of GBS and MFS have been reported following both SARS-CoV-2 infection and, much less frequently, COVID-19 vaccination. The proposed mechanism in both scenarios is molecular mimicry, where antibodies produced against the viral spike protein cross-react with structurally similar gangliosides on peripheral nerves [2]. Recent literature highlights approximately nine total cases of MFS post COVID vaccination [3].

Herein, we report a serologically confirmed case of MFS in a 51-year-old man, detailing his clinical course, diagnostic findings, and functional recovery following the administration of a Pfizer SARS-CoV-2 bivalent mRNA vaccine.

## Case presentation

A 51-year-old previously independent, right-handed, Mandarin-speaking male with a past medical history of GERD, benign prostatic hyperplasia (BPH), chronic right L5 radiculopathy, and right carpal tunnel syndrome presented to the hospital on March 7th, 2025, with five days of progressive neurological symptoms. His history was notable for receiving a Pfizer SARS-CoV-2 bivalent vaccine (2VCOV-mRNA, 30mcg/0.3mL) 10 days prior to symptom onset. He reported no immediate post-vaccination illness.

The patient’s illness began with ascending numbness in his hands and feet. This was followed two days later by progressive ataxia, rendering him unable to sit or stand without assistance. He also developed diplopia on leftward gaze and dizziness upon standing.

On admission, neurological examination revealed an awake and alert patient with significant dysarthria. Cranial nerve assessment was remarkable for a left eyelid ptosis and bilateral facial weakness. He was areflexic in all four limbs. He exhibited severe truncal and appendicular ataxia. He had no bowel or bladder incontinence during his disease course. His respiratory status was monitored closely, with a negative inspiratory force (NIF) of -60 and a vital capacity (VC) of 2.7 L. During his admission, he developed tachycardia and slight variations in blood pressure, suggesting a mild dysautonomia.

An extensive diagnostic workup was performed. Magnetic resonance imaging (MRI) of the brain and entire spine revealed no acute intracranial or spinal cord pathology that could explain his symptoms, showing only chronic degenerative changes consistent with his known history. A lumbar puncture demonstrated the classic finding of cytoalbuminologic dissociation, with a protein level of 61 mg/dL and a white blood cell count of 3 cells/µL. Critically, a serum anti-ganglioside antibody panel, performed on March 6, was positive for anti-GQ1b IgG antibodies, confirming the diagnosis of MFS. Serologies for Lyme disease, syphilis (VDRL), and West Nile virus were negative.

Electromyography (EMG) was performed, which noted subtle electrophysiologic findings compatible with an early acute demyelinating neuropathy, including mildly prolonged right ulnar and tibial F-waves and a prolonged distal CMAP duration in the right peroneal nerve. The study also confirmed his known chronic right L5 radiculopathy and right median mononeuropathy at the wrist.

The patient was treated with a five-day course of intravenous immunoglobulin (IVIg) from March 7 to March 11. He was discharged from the acute care hospital on March 19 and admitted to an inpatient acute rehabilitation facility. There, he received intensive physical, occupational, and speech therapy for three hours a day, five days a week. His respiratory status improved, and his ptosis and diplopia resolved. After exhausting his insurance-covered days at this facility, he still required further therapy to gain more independence, specifically with stair training for his third-story apartment. He was therefore discharged to a sub-acute rehabilitation facility for continued therapy prior to returning home.

## Discussion

This case represents a robust example of MFS, fulfilling the clinical triad [1,2,4], supported by classic CSF findings, and, most importantly, confirmed by the presence of anti-GQ1b antibodies [5]. The serological confirmation is a significant strength of this case, definitively linking the patient's clinical presentation to the known pathophysiology of MFS.

The diagnostic workup successfully excluded critical differential diagnoses. The unremarkable brain and spine imaging ruled out central nervous system mimics like a brainstem stroke or acute demyelinating lesions. The EMG provided objective evidence of a peripheral demyelinating process, distinct from the patient's underlying chronic radiculopathy and carpal tunnel syndrome, which could not account for his acute, symmetrical, and ascending presentation.

The temporal relationship between the Pfizer COVID-19 vaccination and the onset of symptoms - a 10-day latency period - is consistent with the time frame typically observed for other post-vaccination neurological events and post-infectious GBS [6]. While causation cannot be definitively proven from a single case, the absence of any other preceding infectious or immunological trigger makes the vaccination a plausible inciting event through molecular mimicry.

The patient's clinical course, including the development of mild dysautonomia [7] and his positive response to IVIg, is characteristic of MFS [8]. His successful progress through multiple levels of rehabilitation underscores the generally favorable, albeit potentially prolonged, prognosis of this syndrome when identified and treated promptly, similar to other cases of MFS post COVID vaccine [9,10]. This case adds valuable, serologically confirmed evidence to the limited body of literature on the rare but potential association between the Pfizer COVID-19 vaccination and MFS [11,3]. It highlights the importance of recognizing this syndrome and considering it in the differential diagnosis for patients presenting with acute ophthalmoplegia, ataxia, and areflexia following a recent immunological stimulus.

## Conclusions

MFS is a rare but significant neuro-immunological complication of various antecedent triggers. Patients receiving vaccinations may be at a very low but potential risk due to the robust immune activation they induce. This case illustrates how an immune response, plausibly triggered by a bivalent COVID-19 vaccine, can lead to the production of anti-GQ1b antibodies and result in acute, debilitating neurological deficits. Early recognition and timely immunotherapy are crucial for facilitating recovery. This case reinforces the need for vigilance in patients who develop new-onset ataxia and ophthalmoplegia, emphasizing the importance of considering MFS in the differential diagnosis even in the absence of a preceding infection.
